# Breastfeeding Intention and Breastfeeding Postpartum Outcomes between High-Risk and Low-Risk Pregnant Women: A Greek Prospective Cohort Study

**DOI:** 10.3390/ijerph21060755

**Published:** 2024-06-09

**Authors:** Panagiota Brani, Irina Mrvoljak-Theodoropoulou, Fani Pechlivani, Maria Iliadou, Evangelia Antoniou, Georgios Daskalakis, Peter Drakakis, Maria Dagla

**Affiliations:** 1 Department of Midwifery, School of Health & Care Sciences, University of West Attica, 12243 Athens, Greece; pbrani@uniwa.gr (P.B.); fpechliv@uniwa.gr (F.P.); miliad@uniwa.gr (M.I.); lilanton@uniwa.gr (E.A.); 2 Department of Psychology, National & Kapodistrian University of Greece, 15784 Athens, Greece; imrvoljak@hotmail.com; 3 First Department of Obstetrics and Gynecology, Alexandra Hospital, National and Kapodistrian University of Athens, 11528 Athens, Greece; gdaskalakis@med.uoa.gr; 4 Third Department of Obstetrics and Gynecology, University General Hospital “ATTIKON”, Medical School, National and Kapodistrian University of Athens, 12462 Athens, Greece; pdrakakis@med.uoa.gr

**Keywords:** breastfeeding, intention, high-risk pregnancy, exclusive breastfeeding, prospective cohort study

## Abstract

Background: This prospective cohort study, conducted from pregnancy to six months postpartum and grounded in STROBE methodology, quantitatively explores the relationship between antenatal breastfeeding intentions and subsequent breastfeeding outcomes among high-risk pregnant women, compared to a low-risk pregnancy group. Methods: The study was conducted in one of the largest public hospitals in Attica that provides care to pregnant women, enrolling 380 participants divided into high-risk (*n* = 200) and low-risk (*n* = 180) cohorts. Data were collected over 20 months (starting from the end of May 2020 until January 2022), spanning from pregnancy to six months postpartum, via comprehensive questionnaires. Results: Statistical analysis revealed a pronounced correlation between prenatal breastfeeding intentions and actual breastfeeding behaviors across both groups. Specifically, 81.1% of women in the high-risk group and 82.5% in the low-risk group expressed intentions of exclusively breastfeeding during pregnancy. By six months postpartum, 54.9% of the high-risk and 64.3% of the low-risk pregnancy group managed to sustain breastfeeding. Extended antenatal hospitalization emerged as a statistically significant factor (*p* = 0.045) negatively impacting exclusive breastfeeding intentions among high-risk pregnancies. Conclusion: The findings illuminate the critical influence of antenatal intentions on breastfeeding outcomes, particularly among high-risk pregnancies. Moreover, the study identifies the detrimental effect of prolonged hospital stays on breastfeeding aspirations. These insights underscore the necessity for nuanced, supportive interventions aimed at bolstering breastfeeding rates, thereby advancing maternal and neonatal health objectives aligned with World Health Organization recommendations.

## 1. Introduction

Breastfeeding constitutes the “gold standard” for infantile nutritional provisioning, encompassing an array of benefits spanning nutritional, immunological, emotional, economic, and societal dimensions [1,2]. In the short term, it significantly mitigates infant mortality and morbidity attributable to gastrointestinal and respiratory infections, alongside a diminution in the incidence of otitis media [3,4,5,6]. Additionally, breastfeeding is correlated with long-term health dividends, including an augmentation in intelligence quotient (IQ) levels, diminished predisposition towards obesity and type 2 diabetes mellitus, and a lower incidence of childhood leukemia [7,8,9,10,11]. These findings underscore the comprehensive advantages of breastfeeding, highlighting its pivotal role in fostering optimal health outcomes from infancy through to later life stages [12].

For lactating mothers, the act of breastfeeding accelerates uterine involution and diminishes the incidence of postpartum hemorrhage, concurrently affording a protective effect against breast and ovarian carcinomas [13,14]. Breastfeeding represents a critical biological process with salutary implications for maternal physical and emotional well-being during the postnatal period and extending into long-term health [15]. Despite the preponderance of empirical evidence extolling the benefits of breastfeeding and its endorsement by healthcare authorities worldwide, the rates of breastfeeding initiation and the duration thereof exhibit notable variability [16]. This variance is attributable to an amalgam of factors, including individual beliefs, cultural practices, psychological factors, accessibility to supportive resources, and the presence of antenatal medical conditions or complications arising during childbirth. Such multifaceted influences underscore the complexity of breastfeeding practices, necessitating a nuanced approach to promoting lactation across diverse populations [17,18,19].

Suboptimal breastfeeding rates and premature cessation are associated with detrimental health outcomes for both neonates and mothers, contributing to escalated healthcare expenditures and intensifying disparities in health outcomes [20,21,22]. Initiatives aimed at enhancing the initiation rates and prolongation of breastfeeding durations are integral to achieving public health objectives on both national and international fronts [23]. Despite high initiation rates in developed countries, a significant proportion of lactating women terminate breastfeeding prematurely, within the initial weeks or months following parturition. This leads to a disconcertingly low prevalence of breastfeeding at the six-month milestone, a period critical for sustaining exclusive breastfeeding as recommended by global health authorities. This discrepancy highlights the need for targeted interventions to support sustained breastfeeding practices among postpartum women [24,25,26,27,28,29].

Globally, the prevalence of exclusive breastfeeding for infants under six months is reported at 44%, with the rate of breastfeeding at one year reaching 68%. The World Health Organization (WHO) [16] has set forth objectives to elevate the exclusive breastfeeding incidence to 70% at the six-month juncture and to augment the overall breastfeeding prevalence to 80% by the time children reach one year of age by the year 2030. It is advocated that breastfeeding initiation should occur within the inaugural hour following birth, contingent upon maternal intent and the feasibility of breastfeeding [27,30]. Despite the innate capacity of all mothers to breastfeed, contingent on the provision of accurate information and the receipt of comprehensive support from familial, healthcare, and societal systems, the resolution to engage in breastfeeding is profoundly personal [31]. This decision is subject to an array of determinants including but not limited to anticipated impediments, support from healthcare professionals, institutional practices within healthcare facilities, and the potential for prenatal medical complications or challenges encountered during the process of childbirth [32,33,34].

The escalation of complexities encountered by women in the context of pregnancy, attributable to preexisting physical health ailments or conditions manifesting prenatally, amplifies the probability of unfavorable perinatal outcomes, thereby necessitating the provision of specialized obstetric attention. Chronic (physical and mental) health issues or complications arising within the gestational period, inclusive of placental anomalies, Rh factor incompatibility, the premature rupture of the amniotic sac, preterm labor contractions, preeclampsia, intrauterine growth restriction (IUGR), and cervical insufficiency, categorize a gestation as high-risk [35,36,37].

The clinical governance of such conditions and their sequelae may necessitate augmented perinatal or postnatal interventions, potentially impacting postpartum management paradigms, including lactation [38]. While gestations deemed high-risk and their neonates stand to gain from lactational benefits, there exists a minimal spectrum of conditions wherein lactation may not align with the neonate’s optimal health interests. These sparse contraindications notwithstanding, a considerable number of mothers opt against initiating or continuing breastfeeding [39].

In accordance with the Theory of Planned Behavior [40], the intention of mothers to engage in lactation is posited as a direct antecedent to their breastfeeding behaviors and practices. Thus, the antenatal intention to engage in lactation emerges as a pivotal predictor of neonatal nutritive behavior [41]. Yet, the scholarly investigation into the influence of maternal antenatal intentions on the initiation rates and substantive duration of lactation within populations experiencing high-risk pregnancies remains markedly scarce [42]. Internationally, there exists a paucity of research focused on elucidating the factors that may shape maternal intention and subsequent lactational behavior in the aftermath of a high-risk gestational period.

This investigation aims to elucidate the impact of antenatal intentions to engage in lactation among pregnant women undergoing high-risk pregnancies and receiving antenatal care in specialized high-risk obstetric/midwifery units, on their postnatal lactational behaviors, with a particular emphasis on exclusive lactation until the sixth month postpartum. Furthermore, this study intends to examine the effect of specific characteristics inherent to pregnant women with high-risk conditions (e.g., the duration of hospitalization and the intake of medication) on their postnatal aspirations and determination to exclusively breastfeed.

Embarking on an inquiry within a distinct segment of the pregnant population, i.e., those encountering high-risk pregnancies, this research scrutinizes its association with a critical public health concern: breastfeeding outcomes. Notably, in the Greek context, there is an absence of documented evidence regarding the breastfeeding inclinations of pregnant women facing complex pregnancies necessitating antenatal hospitalization in dedicated clinics for high-risk pregnancies. Additionally, the breastfeeding rates postnatally among mothers who have navigated through a high-risk pregnancy remain uncharted in the scientific literature. This pioneering study within the Greek populace endeavors to bridge the identified gap in the literature, serving as an inaugural effort to dissect the factors potentially influencing the breastfeeding intentions of women with high-risk pregnancies who are hospitalized antenatally, alongside assessing how these preconceived intentions shape their breastfeeding behaviors broadly, and specifically towards sustaining exclusive breastfeeding through the first six months postpartum.

The dearth of information on the breastfeeding intentions and outcomes within this particular cohort underscores a notable lacuna in comprehending the extensive range of breastfeeding challenges and possibilities. High-risk pregnancies, delineated by augmented medical intricacies and the requisite for specialized medical oversight, introduce distinctive impediments to the initiation and continuation of breastfeeding. Such pregnancies might encompass conditions that potentially disrupt or impede breastfeeding endeavors, including but not limited to prematurity, maternal health complications, and the immediate medical necessities of the infant subsequent to birth. Consequently, the probe into the breastfeeding intentions and behaviors of women enduring high-risk pregnancies in Greece not only endeavors to shed light on the experiences and outcomes pertinent to this specific group but also aims to enrich the overarching dialogue on maternal and infant health. This research, by filling the existent void, is poised to influence health care policies and practices to be more inclusive of the nuanced needs of mothers confronted with the supplementary burdens of a high-risk pregnancy, thereby aiding these women in realizing their lactational objectives. The objective of this study is to investigate the impact of antenatal breastfeeding intentions on postnatal breastfeeding behaviors, particularly exclusive breastfeeding up to six months postpartum, among women experiencing high-risk pregnancies and receiving specialized antenatal care.

## 2. Materials and Methods

### 2.1. Study Design and Objectives

Aligned with the guidelines set forth by the STROBE (Strengthening the Reporting of Observational Studies in Epidemiology) statement, this investigation was strategically designed as a prospective cohort study. Its principal aim centered on examining breastfeeding intentions (whether exclusive or non-exclusive breastfeeding) and the anticipated duration of breastfeeding among pregnant women identified as high-risk, who were under the care of a specialized clinic for high-risk pregnancies. Moreover, the study sought to delve into the determinants that influence these breastfeeding intentions and to meticulously document breastfeeding outcomes, with a particular emphasis on the woman’s behavior towards exclusive breastfeeding through the first six months postpartum. It is pertinent to note that the study site lacks current certification as a Baby-Friendly Hospital according to UNICEF-WHO standards. Certification efforts were temporarily suspended due to the COVID-19 pandemic and the resultant implementation of restrictive measures. Likewise, the Neonatal Intensive Care Unit (NICU) at the study site is not designated as a Baby-Friendly NICU. Nevertheless, breastfeeding counseling and support are consistently provided across all hospital departments. This continuous support highlights the hospital’s commitment to promoting breastfeeding practices, despite the absence of formal certifications.

### 2.2. Setting and Participants

The study was conducted from pregnancy to six months postpartum in one of the largest public hospitals in Attica that provides care to pregnant women. This facility stands as one of the preeminent general public hospitals with obstetric clinics in Greece, chosen for its extensive annual birth rate and its comprehensive provision of specialized care for pregnancies deemed high-risk. This choice of location adheres to the directives provided by the STROBE guidelines, which advocate for an explicit delineation of the research setting.

To delineate a well-defined study cohort, eligibility criteria were meticulously established. These criteria stipulated that participants must be women aged 18 years or older, possess proficiency in the Greek language, and have completed childbirth at the aforementioned hospital, with a gestational age of 32 weeks or more. The establishment of these precise criteria was instrumental in curating a homogeneous study population, thereby substantially augmenting the reliability and validity of the derived research outcomes.

### 2.3. Sample Size and Sampling Technique

The initial recruitment encompassed two distinct cohorts: 200 pregnant women classified within the high-risk category and 180 women identified as constituting a low-risk pregnancy group. This delineation was predicated on a priori sample size calculations designed to secure adequate statistical power for the investigation. To ensure comparability, the low-risk pregnancy group was selected from the same hospital, with efforts made to match the proportion of high-risk and low-risk pregnancies based on hospital admission data. The methodology employed for sampling, coupled with the documented response rates—82% for the high-risk group and 85% for the low-risk group—were meticulously chronicled. Our final groups consisted of 164 women of high-risk pregnancy and 154 women of low-risk pregnancy. Such adherence underscores the study’s commitment to methodological transparency and the facilitation of reproducibility in future research endeavors.

### 2.4. Data Collection

The data collection period for this study spanned 20 months, starting from the end of May 2020 until January 2022, methodically partitioned into five distinct phases. This stratification was meticulously designed to systematically monitor the trajectory of breastfeeding intentions and actual practices from the prenatal period through to the sixth month following childbirth. Adopting this segmented approach facilitated a thorough longitudinal examination of breastfeeding behaviors, in strict accordance with the STROBE guidelines which advocate for the explicit delineation of research timelines.

The initiation of the study incorporated a pilot phase, instrumental in fine-tuning the investigative instruments and methodology, thereby ensuring the integrity and reliability of the data gathered. The ensuing phases embraced a hybrid model of data collection methodologies, encompassing both direct interactions (face-to-face) and indirect engagements (via telephone and digital submissions through Google Forms). This methodological pluralism was deliberately chosen to accommodate the varied preferences of the participants, thereby optimizing participation rates and enhancing the comprehensiveness of the dataset.

### 2.5. Ethical Considerations

In alignment with the ethical standards prescribed by the Declaration of Helsinki and adhering to stringent ethical scrutiny, this study underwent a thorough review process and received formal approval from the respective Institutional Review Boards (IRBs) before initiation. This procedural step ensured that the study’s design, methodology, and participant interaction protocols conformed to established ethical guidelines and principles for research involving human subjects. The application submitted to the scientific council was assigned the protocol number 346 on 20 May 2020. Approval was granted during the 6th meeting of the council, which took place on 26 May 2020. This approval was essential for the commencement of the study, ensuring that all ethical considerations and research protocols were rigorously reviewed and met the council’s standards before the initiation of data collection.

Prior to participation, all potential subjects received detailed information about the study’s objectives, methods, risks, and benefits, enabling informed consent. Participants were assured of their voluntary involvement, with the freedom to withdraw at any time without consequences. The consent form emphasized confidentiality, detailing measures to anonymize data and restrict access. Additionally, the form outlined protocols for secure data handling and storage, ensuring ethical adherence and respect for participant rights throughout the study.

### 2.6. Research Questionnaires 

To meticulously capture the multifaceted nature of breastfeeding intentions and outcomes among the study population, a series of bespoke, anonymized questionnaires was meticulously constructed. These questionnaires were intricately designed to systematically gather comprehensive data across various dimensions, including socio-demographic attributes, detailed obstetric history, explicit breastfeeding intentions, and the subsequent actualization of these breastfeeding practices postpartum. The construction of these questionnaires was underpinned by an exhaustive review of the relevant literature and existing validated questionnaires, ensuring their alignment with the study’s intricate objectives and the overarching research questions it sought to address.

The deployment of these questionnaires was strategized across multiple phases of the study, each tailored to capture data pertinent to distinct temporal stages—ranging from prenatal intentions through to breastfeeding practices up to six months post delivery. This phased approach allowed for the longitudinal tracking of breastfeeding behaviors and the identification of potential shifts in intentions and practices over time. In developing these research questionnaires, significant emphasis was placed on ensuring their reliability and validity. This involved a rigorous pilot testing phase, where a subset of the target population was engaged to identify potential ambiguities or biases in the questions. Feedback obtained during this phase was critically analyzed and used to refine the questionnaires, enhancing their clarity and effectiveness in eliciting accurate and meaningful responses.

Additionally, the questionnaires incorporated a blend of closed-ended and open-ended questions. This combination was employed to not only facilitate ease of analysis through quantifiable data but also to capture nuanced insights and personal experiences that might not be easily quantified. The open-ended questions, in particular, were instrumental in providing depth to the quantitative data, allowing participants to articulate their experiences, perceptions, and challenges related to breastfeeding in their own words. The administration of these questionnaires was conducted with utmost consideration for the participants’ convenience and preference, utilizing a mix of direct (in-person) and indirect (telephone, online via secured platforms) methods. This flexibility in data collection methodology not only maximized participant response rates but also catered to the diverse needs and circumstances of the study population, ensuring broad and inclusive participation across the spectrum of high-risk pregnancies.

This study was conducted in five distinct phases, each designed to capture critical data at various stages of the perinatal and postnatal period. 

Phase 1: During the initial phase, both high-risk pregnant women during their prenatal hospitalization in the high-risk pregnancy unit and low-risk pregnant women attending regular outpatient clinics completed a series of questionnaires. These included socio-demographic and medical characteristics. 

Phase 2: The second phase took place on the 3rd to 4th day postpartum, during the participants’ stay in the postnatal wards. Questionnaires administered included a follow-up on feeding methods post delivery.

Phase 3: At the end of the puerperium, further assessments were conducted through phone interviews or a specially designed online questionnaire form, including the outcome of breastfeeding practices.

Phase 4: Three months postpartum, the fourth phase involved collecting data through phone interviews or electronic forms, assessing ongoing breastfeeding status. 

Phase 5: At the six-month postpartum period, final assessments were conducted to evaluate the long-term outcomes of breastfeeding practices.

Each phase was carefully planned to provide a comprehensive understanding of the breastfeeding journey, from prenatal intentions to postnatal practices, particularly focusing on the unique challenges faced by women with high-risk pregnancies.

### 2.7. Data Analysis

The definitions of breastfeeding provided by the WHO are crucial for ensuring consistency and comparability in breastfeeding research and practices globally. According to WHO guidelines, breastfeeding should commence within the first hour of life. Exclusive breastfeeding is recommended for the first six months of life, meaning that the infant receives only breast milk without any additional food or drink, not even water, except for oral rehydration solution, drops, or syrups consisting of vitamins, minerals, or medicines. The term ‘mixed feeding’ or ‘partial breastfeeding’ refers to feeding both breast milk and other foods or liquids. Continued breastfeeding, with appropriate complementary foods, is recommended for up to two years of age or beyond. These standardized definitions allow for the meaningful comparison of breastfeeding rates across different studies and populations, ensuring that data on breastfeeding practices are reliably measured and interpreted universally [43,44].

In the rigorous examination of the collected dataset, a statistical analysis was executed employing the Statistical Package for the Social Sciences (SPSS), version 22.0. The analytical methodology was bifurcated to adeptly handle the distinct nature of categorical and continuous variables within the dataset. For categorical variables, encompassing binary or nominal data reflecting classifications such as breastfeeding intention status, type of delivery, and demographic categorizations, a detailed frequency analysis was conducted. This involved calculating the percentages and frequencies of each category, thereby elucidating the distribution patterns of these variables within the study population. Such an approach provides a clear snapshot of the prevalence and distribution of key characteristics and behaviors among the participants.

In parallel, continuous variables, which include data points that represent measurements on a continuous scale such as age, the duration of breastfeeding, and gestational age at delivery, were analyzed through the computation of means and ranges. This statistical treatment enabled the capture of central tendencies and the variability within these measurements, offering insights into the average behaviors and the spread of data points around these averages. This method is particularly valuable in identifying the central trends within the data, facilitating a nuanced understanding of the study outcomes. Furthermore, the analytical process included a series of comparative analyses, tests such as Fisher’s Exact Test for categorical variables, and Binary logistic regression analysis. This enabled the exploration of significant differences and relationships between various groups within the study population, such as comparing breastfeeding intentions and outcomes between high-risk and low-risk pregnancy cohorts.

The strategic application of this comprehensive statistical analysis not only ensures the reliability and validity of the study’s findings but also enhances their applicability and comparability with the existing literature. By adhering to the rigorous standards set forth by the STROBE guidelines and employing a robust statistical methodology, this study contributes valuable empirical insights into the breastfeeding intentions and behaviors of women with high-risk pregnancies, paving the way for further research and informed clinical practice in this critical area of maternal and child health.

## 3. Results

The characterization of the socio-demographic and perinatal variables within the study cohort is meticulously detailed in Table 1, which delineates the distinctions between the high-risk pregnancy group and low-risk pregnancy group. An examination of age demographics reveals a mean age of 33.75 years (*Standard Deviation* [*SD*] = 5.48) within the high-risk pregnancy group cohort, compared to a slightly lower mean age of 31.69 years (*SD* = 6.00) among the low-risk pregnancy group participants. Educational attainment across both cohorts indicates a substantial proportion of university graduates, comprising 44.5% (*n* = 73) of the high-risk pregnancy group and 48.1% (*n* = 74) of the low-risk pregnancy group, suggesting a relatively high level of educational background among participants. Marital status distribution reveals a predominance of married individuals within both groups, accounting for 83.5% (*n* = 137) of the high-risk pregnancy group and 90.9% (*n* = 140) of the low-risk pregnancy group. This demographic characteristic underscores the socio-familial context within which the perinatal decisions and experiences of these women are situated. Focusing on perinatal history, the mode of delivery presents notable differences between the groups. The high-risk pregnancy group exhibits a significantly higher prevalence of caesarean section deliveries, constituting 79.9% (*n* = 131) of births, in stark contrast to the low-risk pregnancy group, where caesarean sections account for 48.1% *(n* = 74) of deliveries. This disparity highlights the potential influence of high-risk pregnancy conditions on the mode of delivery decision-making process. 

The distribution of parity within the sample indicates that for the majority of the women, the current pregnancy culminated in their first child, with 54.3% (*n* = 89) in the high-risk pregnancy group and 56.5% (*n* = 87) in the low-risk pregnancy group. This demographic feature is critical for understanding the participants’ perinatal experiences and their implications for breastfeeding intentions and practices. Regarding breastfeeding decision timing, a substantial majority across both groups reported making their breastfeeding decision prior to pregnancy, with 76.2% (*n* = 125) in the high-risk pregnancy group and 81.8% (*n* = 126) in the low-risk pregnancy group. This finding suggests a high level of pre-pregnancy contemplation and planning concerning infant feeding choices among the participants. Finally, the gestational age at birth presents a pronounced contrast between the two groups. In the high-risk pregnancy group, a significant proportion of women (27.4%, *n* = 45) underwent childbirth at or before the 37th completed week of gestation, indicating preterm births, as opposed to a mere 2.6% (*n* = 4) within the low-risk pregnancy group. This stark variance underscores the heightened perinatal risk profile of the high-risk pregnancy group, potentially implicating the prenatal and postnatal care strategies, including breastfeeding initiation and sustainability.

In Table 2, it can be observed that an analogous percentage of pregnant women from the high-risk pregnancy group (*n* = 133, 81.1%) and low-risk pregnancy group (*n* = 127, 82.5%) during the pregnancy intended to breastfeed exclusively. Subsequently, 54.9% of women from the high-risk pregnancy group (*n* = 90) and 64.3% from the low-risk pregnancy group (*n* = 99) breastfed for more than six months.

Table 3 presents the results of Fisher’s Exact Test analyses of the pregnant woman’s desire to breastfeed in relation to the way she finally chose to feed her child. As can be seen, during the measurement time phases, statistically significant results are recorded: (a) in the 1st 24 h postpartum (Fisher’s Exact Test = 9.607, *df* = 2, *p* = 0.016), only in the low-risk pregnancy group; (b) in the 3rd 24 h, in both groups (high-risk pregnancy group—Fisher’s Exact Test = 9.032, *df* = *2*, *p* = 0.022; low-risk pregnancy group—Fisher’s Exact Test = 10.358, *df* = 2, *p* = 0.005); (c) at the 6th week postpartum, in both groups (high-risk pregnancy group—Fisher’s Exact Test = 12.771, *df =* 2, *p* = 0.001; low-risk pregnancy group—Fisher’s Exact Test = 23.211, *df* = 2, *p* < 0.001), (d) at the 3rd month postpartum, in both groups (high-risk pregnancy group—Fisher’s Exact Test = 11.294, *df =* 2, *p* = 0.001; low-risk pregnancy group—Fisher’s Exact Test = 18.876, *df =* 2, *p* < 0.001); and (e) at the 6th month postpartum, in both groups (high-risk pregnancy group—Fisher’s Exact Test = 10.512, *df* = 2, *p =* 0.004; low-risk pregnancy group—Fisher’s Exact Test = 13.980, *df* = 2, *p* = 0.001). Pregnant women who intended to breastfeed exclusively appeared to choose exclusive breastfeeding to a greater extent, in all the above measurement time phases, in comparison to pregnant women who intended to breastfeed non-exclusively. It should be mentioned that there was no statistically significant difference between the intention of the pregnant women to breastfeed and the way of feeding the newborn in the 1st 24 h, when it comes to the high-risk pregnancy group.

Regarding the relationship between the intention of the pregnant woman to breastfeed in relation to the number of her children (Table 4), a statistically significant difference emerged only in the low-risk pregnancy group (Fisher’s Exact Test = 5.713, *df* = 2, *p* = 0.050). A greater desire for exclusive breastfeeding, in relation to non-exclusive breastfeeding, seems to exist for their second child. Furthermore, as far as the sample of the present research is concerned, an insignificant number of women choose non-exclusive breastfeeding for their 3rd/>3rd child. 

The results of the analysis, through Fisher’s Exact Test, regarding the relationship between the intention to breastfeed and whether the pregnancy was planned or not, are presented in Table 5. From the analysis, a statistically significant relationship emerged between the intention to breastfeed and the pregnancy type only in the high-risk pregnancy group (Fisher’s Exact Test = 8.151, *df* = 2, *p* = 0.012). Τhere was a greater intention of exclusively breastfeeding in the cases where the pregnancy was planned, compared to unplanned pregnancy and after assisted reproduction.

Table 6 presents the results of the Fisher’s Exact Test analysis regarding the relationship between the pregnant woman’s intention to breastfeed and the time frame when she made such decision. The results of the analysis show a statistically significant relationship between the intention to breastfeed and the period this decision was made only in the high-risk pregnancy group (Fisher’s Exact Test = 7.153, *df* = 2, *p* = 0.020). The pregnant woman’s greater intention of exclusively breastfeeding, in relation to the intention of non-exclusively breastfeeding, seems to occur when the relevant decision is made before pregnancy.

From the next analysis (Table 7), a statistically significant relationship appeared between the pregnant woman’s intention to breastfeed and breastfeeding difficulties only in the low-risk pregnancy group, and only at the 6th week postpartum (Fisher’s Exact Test = 16.444, *df* = 2, *p* = 0.001). It seems that pregnant women in the low-risk pregnancy group who wished to exclusively breastfeed did not appear to experience difficulties with breastfeeding at the 6th week postpartum, or they experienced them less than expected, in comparison to those who planned non-exclusive breastfeeding, whereas the opposite results were recorded. 

Finally, binary logistic regression analysis was applied (Table 8) to examine the relationship between the woman’s (high-risk pregnancy) intention of exclusively breastfeeding and the variables related to her (a) satisfaction with the current pregnancy, (b) medication intake, and (c) days of hospitalization, during the period of the current pregnancy. The results show that, out of the analyzed possible predictors, only the hospitalization of the pregnant woman for a period of more than 15 days appears as a predictor (*p* = 0.045) for the high-risk pregnancy group desire for exclusive breastfeeding. This model explains 8.1% of the total variance of the dependent variable. As it turns out, pregnant women who belonged to the high-risk pregnancy group and had been hospitalized for more than 15 days during pregnancy, compared to those who had been hospitalized for fewer days, had a lower intention of exclusively breastfeeding after delivery.

## 4. Discussion

This investigation delineates the continuum of breastfeeding practices, initiating from maternal predispositions towards breastfeeding during the gestational phase through to exclusive breastfeeding upon hospital discharge, extending to the sixth month postpartum. It encapsulates preliminary findings from an explorative study situated in Greece, scrutinizing both the determinants and eventualities of breastfeeding, with an acute focus on exclusive breastfeeding practices until the sixth month postpartum among a demographically specific cohort—women navigating complex, high-risk pregnancies necessitating specialized prenatal interventions within a designated high-risk pregnancy care unit.

The paramountcy of breastfeeding as the optimal nutritional modality for neonates is well documented, underpinned by a robust corpus of research delineating its multifaceted benefits for maternal and neonatal health alike [34,45]. Prenatal medical risks have been associated with breastfeeding outcomes up to 12 months, as indicated by Scime et al. [46], while Lyons et al. [47] highlight that breast milk is a critical source of beneficial microbes, offering substantial health benefits to infants. The maternal predilection towards exclusive breastfeeding, or, alternatively, an amalgamated approach integrating formula feeding, emerges as a formidable predictor of both the initiation and sustained practice of breastfeeding [48,49,50]. In the ambit of this study, the predictive value of maternal intentions towards breastfeeding subsequent to a high-risk pregnancy trajectory was substantiated as a significant determinant for both the commencement and the subsequent continuity of breastfeeding practices [51,52,53,54]. Call et al.’s [55] longitudinal research examines breastfeeding planning, initiation, and duration among individuals with pre-pregnancy overweight or obesity, revealing that these individuals often face unique challenges and lower breastfeeding rates compared to those with normal weight. Nonetheless, the endeavor to juxtapose findings across diverse studies encounters methodological impediments, primarily attributable to the absence of a universally standardized definition encapsulating the initiation of breastfeeding [56]. The WHO has operationalized breastfeeding categorization to include infants receiving “a single daily feeding of breast milk or any breastfeeding attempt preceding their hospital discharge” [54,55,56,57,58,59].

The prevalence of breastfeeding intentionality within our cohort exhibited parity across the dichotomy of high-risk (81.1%) and low-risk (82.5%) pregnancy groups. This parity resonates with the findings of Cordero et al. [51,52]. Moreover, a more recent research study noted that severe preeclampsia is associated with lower breastfeeding initiation rates [60]. Conversely, a review of the extant literature reveals a predilection towards diminished exclusive breastfeeding intentionality rates within high-risk pregnancy contexts when juxtaposed to low-risk scenarios, a pattern not mirrored within the purview of our investigation. This divergence underscores the imperative for a nuanced understanding and interrogation of the multifactorial influences modulating breastfeeding intentions and practices, especially within high-risk pregnancy demographics. The elucidation of these dynamics is quintessential for the crafting and implementation of targeted interventions and policies aimed at optimizing breastfeeding practices. Thus, this study contributes a novel perspective to the existing body of knowledge, paving the way for subsequent empirical inquiries and the formulation of evidence-based public health strategies designed to foster and sustain breastfeeding among diverse maternal populations [34,51,54].

The investigation conducted by Diaz et al. [39] elucidates a nuanced relationship between the rationale for referral to specialized high-risk obstetric care and maternal breastfeeding plans. Specifically, an association was identified exclusively among cohorts referred due to the peril of preterm delivery, wherein a diminished propensity towards breastfeeding planning was observed. Strapasson et al. [61] further reinforce this notion, highlighting that gestational hypertension negatively impacts feeding practices in the first 6 months postpartum, with affected women showing lower rates of breastfeeding initiation and continuation. This trend underscores the criticality of the underlying reason for high-risk categorization in influencing maternal breastfeeding intentions. Furthermore, it is noteworthy that women encountering high-risk pregnancies who, during the prenatal phase, articulate a disinclination towards breastfeeding, opting instead for formula feeding for their offspring, seldom deviate from this initial decision postnatally [51,52,53,54].

Consistent with the literature, the present study underscores a dichotomy in breastfeeding outcomes based on prenatal intentions. Women harboring a prenatal inclination towards exclusive breastfeeding manifested a significantly lower likelihood of cessation when compared to their counterparts favoring a combination of breastfeeding and formula feeding. Huang et al. [62] identify several predictive factors for exclusive breastfeeding attrition at week 6 postpartum among mothers of preterm infants, including maternal confidence and perceived behavioral control, which significantly influence the continuation of exclusive breastfeeding. This empirical observation is corroborated by a constellation of studies, thereby reinforcing the predictive validity of prenatal breastfeeding intentions on postnatal breastfeeding practices [34,50,53,63,64].

Intriguingly, this study unveils a negative correlation between the span of antenatal hospitalization in a high-risk pregnancy milieu and the fervor for exclusive breastfeeding. Specifically, our findings reveal that women subjected to prolonged hospital stays exceeding 15 days during pregnancy exhibited a diminished predilection for exclusive breastfeeding in the postpartum period relative to those with shorter durations of hospitalization. This association, hitherto unexplored in the existing body of research, suggests a potential impact of extended prenatal hospitalization on maternal breastfeeding intentions and preferences.

Furthermore, the timing of the decision-making process regarding infant feeding modalities emerged as a pivotal determinant of breastfeeding intention. The predilection for exclusive breastfeeding was markedly higher among women who determined their feeding strategy prior to conception, compared to those opting for mixed feeding modalities. Additionally, the study delineates a statistically significant nexus between the antenatal resolution to exclusively breastfeed and subsequent breastfeeding outcomes at critical junctures postpartum—namely, at the culmination of the puerperium, as well as at the 3rd and 6th months [49,63,65]. This alignment with existing scholarly discourse underscores the prognostic value of antenatal breastfeeding intentions as robust indicators of breastfeeding initiation and persistence. Notably, our analysis did not discern a statistically significant disparity in the breastfeeding inclinations of women with high-risk pregnancies vis à vis the neonatal feeding regimen within the inaugural 24 h postnatal window. This observation resonates with broader research findings, which suggest that breastfeeding initiation rates among mothers navigating high-risk pregnancies generally trail those observed within the general populace. This lag can be primarily attributed to the higher incidence of immediate postnatal neonatal unit admissions among offspring of high-risk pregnancies, thereby impinging upon the timely initiation of breastfeeding practices [51,52,53,58,63].

This exploration contributes to the scientific discourse by articulating the intricate interplay between antenatal maternal intentions, high-risk pregnancy dynamics, and breastfeeding outcomes. It underscores the imperative for nuanced, context-sensitive support strategies tailored to the unique needs of women undergoing high-risk pregnancies, thereby fostering conducive environments for the realization of breastfeeding intentions and enhancing neonatal nutritional outcomes. The elucidation of these empirical findings necessitates an intensified focus on investigative efforts aimed at unraveling the myriad factors that sculpt maternal intentions towards breastfeeding, particularly in the milieu of high-risk pregnancies. There exists a critical imperative to delve into the multifaceted determinants that modulate breastfeeding intentions and actual practices post high-risk gestational experiences, with the objective of cultivating a robust corpus of empirical evidence. Such scholarly endeavors are instrumental in transcending the realm of ambiguous statistical representations, thereby furnishing a granular understanding of the intricate dynamics at play.

This analytical pursuit aligns with the broader scientific quest to distill actionable insights that can inform targeted interventions and policy formulations. By meticulously examining the interplay between prenatal intentions, high-risk pregnancy parameters, and subsequent breastfeeding behaviors, research can illuminate pathways to bolster breastfeeding rates among this vulnerable cohort. The overarching goal is not only to elucidate the predictors of breastfeeding intentionality and success within the high-risk pregnancy spectrum but also to harness these insights to engineer supportive frameworks that facilitate the realization of breastfeeding aspirations. In essence, advancing this domain of maternal and child health research embodies a pivotal step towards optimizing breastfeeding practices, a cornerstone of neonatal nutrition and maternal well-being. Through a systematic and rigorous examination of the antecedents and correlates of breastfeeding intentions and outcomes in the context of high-risk pregnancies, the scientific community can contribute to the development of a nuanced, evidence-based understanding. This, in turn, holds the potential to significantly impact public health strategies, ensuring that they are meticulously tailored to meet the unique needs of women navigating the complexities of high-risk gestational trajectories, thereby enhancing the overall health outcomes for both mothers and their offspring.

The limitations of this study include its single-hospital setting, potentially limiting the generalizability of the results. The reliance on self-reported data may introduce recall and social desirability biases. The participant sample was not very diverse, primarily consisting of educated, married women, which might not reflect the broader population of pregnant women. Additionally, the study did not explore all possible influences on breastfeeding, such as psychosocial factors and partner support. The observational design also limits the ability to determine causality between the factors studied and breastfeeding outcomes.

## 5. Conclusions

This investigation elucidates the critical role of maternal predilection towards exclusive and prolonged breastfeeding, affirming the association between the anticipatory intentions of women encountering high-risk pregnancies and the subsequent realization of higher incidences of exclusive maternal breastfeeding up to the sixth month postpartum. The elucidation of prenatal breastfeeding predispositions among this demographic, coupled with an in-depth exploration of the determinants shaping such intentions and their consequent behaviors towards breastfeeding, is indispensable for the provision of nuanced support to these individuals, facilitating their navigation through potential lactational adversities. The empirical evidence derived from this study enriches the scientific corpus, offering invaluable insights for midwives and healthcare practitioners tasked with the care and support of women categorized within this “vulnerable” cohort during gestation. Moreover, these insights have direct implications for the spectrum of care, encompassing counseling, educational initiatives, and guidance throughout the breastfeeding continuum. The proactive identification and engagement of women undergoing high-risk pregnancies—who are predisposed to abstain from initiating breastfeeding or to terminate it prematurely owing to the cumulative burden of antenatal complications, extensive hospitalization periods, and apprehensions surrounding the likelihood of preterm delivery—emerge as a pivotal strategy within the midwifery domain, aimed at fostering maternal breastfeeding practices within units specializing in high-risk pregnancies.

Hence, the revelations of this study serve as a foundational pillar for the conceptualization and execution of bespoke interventions and strategies, both antenatally and postnatally, targeted at this increasingly prevalent segment of the pregnant population. Such strategic endeavors are poised to ameliorate initiation rates and the longevity and exclusivity of maternal breastfeeding practices, ultimately striving to reconcile existing discrepancies between prevailing methodologies and the WHO’s breastfeeding directives, with the overarching aim of optimizing health trajectories for both mothers and their progeny.

## Figures and Tables

**Table 1:** Demographic, perinatal, and breastfeeding characteristics.

Demographic Characteristics	High-Risk Pregnancy Group		Low-Risk Pregnancy Group	
	*N/M*	*%/SD*	*N/M*	*%/SD*
Age	33.75	5.48	31.69	6.00
Education				
Primary School	17	10.4	6	3.9
High School	51	31.1	53	34.4
Bachelor’s Degree	73	44.5	74	48.1
Master’s/PhD	23	14.0	21	13.6
Total	164	100.0	154	100.0
Marital Status				
Married	137	83.5	140	90.9
Single	16	9.8	9	5.8
Divorced/Separated	2	1.2	-	-
Partnership Agreement	9	5.5	5	3.2
Total	164	100.0	154	100.0
Number of Children				
1	89	54.3	87	56.5
2	52	31.7	53	34.4
≥3	23	14.0	12	7.8
Total	164	100.0	152	98.7
Perinatal Characteristics				
Type of Delivery				
Vaginal	33	20.1	80	51.9
Caesarian section	131	79.9	74	48.1
Total	164	100.0	154	100.0
Time frame for making the decision to breastfeed				
Before pregnancy	125	76.2	126	81.8
In pregnancy/Postpartum	39	23.8	28	18.2
Total	164	100.0	154	100.0
Week of Labor Onset				
≥37th	119	72.6	150	97.4
<37th	45	27.4	4	2.6
Total	164	100.0	150	97.4

**Table 2:** Intention of the pregnant woman to breastfeed and breastfeeding duration.

		High-Risk Pregnancy Group		Low-Risk Pregnancy Group	
		*N*	*%*	*N*	*%*
Intention of the pregnant woman to breastfeed	Exclusive Breastfeeding	133	81.1	127	82.5
	Non-exclusive Breastfeeding	26	15.9	25	16.2
	Total 1	159	97.0	152	98.7
	Missing values	5	3.0	2	1.3
	Total 2	164	100.0	154	100.0
Breastfeeding duration	≤6 months	67	40.9	55	35.7
	>6 months	90	54.9	99	64.3
	Total 1	157	95.7	154	100.0
	Missing values	7	4.3	-	-
	Total 2	164	100.0	-	-

**Table 3:** Fisher’s Exact Test of the pregnant woman’s intention to breastfeed in relation to the way she chose to feed her child.

		Breastfeeding in the 1st 24 h Postpartum					
		*N*					
Intended to breastfeed			Exclusive Breastfeeding	Only Formula	Non-exclusive Breastfeeding	Fisher’s Exact Test	*p*
Low-risk Pregnancy Group	Exclusive Breastfeeding	O.	64	14	48	9.607	0.016
		E.	58.5	18.4	49.3		
	Non-exclusive Breastfeeding	O.	6	8	11		
		E.	11.5	3.6	9.7		
		**Breastfeeding in the 3rd 24 h postpartum**					
High-risk Pregnancy Group	Exclusive Breastfeeding	O.	40	16	67	9.032	0.022
		E.	36.0	20.9	66.9		
	Non-exclusive Breastfeeding	O.	3	9	13		
		E.	7.0	4.1	13.1		
Low-risk Pregnancy Group	Exclusive Breastfeeding	O.	84	5	38	10.358	0.005
		E.	76.9	5.8	44.3		
	Non-exclusive Breastfeeding	O.	8	2	15		
		E.	15.1	1.2	8.7		
		**Breastfeeding at the 6th week postpartum**					
High-risk Pregnancy Group	Exclusive Breastfeeding	O.	72	31	30	12.771	0.001
		E.	64.4	37.6	30.9		
	Non-exclusive Breastfeeding	O.	5	14	7		
		E.	12.6	7.4	6.1		
Low-risk Pregnancy Group	Exclusive Breastfeeding	O.	87	14	26	23.211	<0.001
		E.	76.9	14.2	35.9		
	Non-exclusive Breastfeeding	O.	5	3	17		
		E.	15.1	2.8	7.1		
		**Breastfeeding at the 3rd month postpartum**					
High-risk Pregnancy Group	Exclusive Breastfeeding	O.	65	45	23	11.294	0.001
		E.	56.9	51.0	25.1		
	Non-exclusive Breastfeeding	O.	3	16	7		
		E.	11.1	10.0	4.9		
Low-risk Pregnancy Group	Exclusive Breastfeeding	O.	79	28	20	18.876	<0.001
		E.	69.3	33.4	24.2		
	Non-exclusive Breastfeeding	O.	4	12	9		
		E.	13.7	6.6	4.8		
		**Breastfeeding at the 6th month postpartum**					
High-risk Pregnancy Group	Exclusive Breastfeeding	O.	58	56	19	10.512	0.004
		E.	51.0	61.1	20.9		
	Non-exclusive Breastfeeding	O.	3	17	6		
		E.	10.0	11.9	4.1		
Low-risk Pregnancy Group	Exclusive Breastfeeding	O.	76	37	14	13.980	0.001
		E.	67.7	42.6	16.7		
	Non-exclusive Breastfeeding	O.	5	14	6		
		E.	13.3	8.4	3.3		

Note. O.—Observed, E.—Expected.

**Table 4:** Fisher’s Exact Test of the pregnant woman’s intention to breastfeed in relation to the number of her children.

		Number of Children					
		*N*					
Intended to breastfeed			1st child	2nd child	3rd/ >3rd child	Fisher’s Exact Test	*p*
Low-risk Pregnancy Group	Exclusive Breastfeeding	O.	67	48	10	5.713	0.050
		E.	72.5	43.3	9.2		
	Non-exclusive Breastfeeding	O.	20	4	1		
		E.	14.5	8.7	1.8		

Note. O.—Observed, E.—Expected.

**Table 5:** Fisher’s Exact Test of the pregnant woman’s intention to breastfeed in relation to pregnancy planning.

		Pregnancy Planning					
		*N*					
Intended to breastfeed			Planned pregnancy	Unplαnned pregnancy	After assisted reproduction	Fisher’s Exact Test	*p*
High-risk Pregnancy Group	Exclusive Breastfeeding	O.	87	40	6	8.151	0.012
		E.	81.1	43.5	8.4		
	Non-exclusive Breastfeeding	O.	10	12	4		
		E.	15.9	8.5	1.6		

Note. O.—Observed, E.—Expected.

**Table 6:** Fisher’s Exact Test of the pregnant woman’s intention to breastfeed in relation to the time frame of making that decision.

		Time Frame for Making the Decision to Breastfeed				
		*N*				
Intended to breastfeed			Before pregnancy	In pregnancy/ Postpartum	Fisher’s Exact Test	*p*
High-risk Pregnancy Group	Exclusive Breastfeeding	O.	107	26	7.153	0.020
		E.	102.1	30.9		
	Non-exclusive Breastfeeding	O.	15	11		
		E.	19.9	6.1		

Note. O.—Observed, E.—Expected.

**Table 7:** Fisher’s Exact Test of the pregnant woman’s intention to breastfeed in relation to the difficulties they faced with breastfeeding as mothers.

			Breastfeeding Difficulties at the 6th Week Postpartum							
			*N*							
Intention to breastfeed				Not at all	A little bit	Moderate	Quite much	Very much	Fisher’s Exact Test	*p*
Low-risk Pregnancy Group	Exclusive Breastfeeding		O.	80	56	41	44	30	16.444	0.001
			E.	68.8	56.2	45.3	47.8	32.7		
	Non-exclusive Breastfeeding		O.	2	11	13	13	9		
			E.	13.2	10.8	8.7	9.2	6.3		

Note. O.—Observed, E.—Expected.

**Table 8:** Binary logistic regression analysis model of the woman’s (high-risk pregnancy) intention of exclusively breastfeeding antenatally (as dependent variable) in relation to hospitalization days, medication intake, and pregnancy satisfaction.

	Woman’s Intention of Exclusively Breastfeeding (Antenatally)				
High-Risk Pregnancy Group	*B*	*S.E.*	*p*	*Exp(B)*	*R* ^2^
Medication intake	−0.711	0.516	ns	0.491	0.081
Pregnancy satisfaction	0.106	0.594	ns	1.112	
Hospitalization days (4 to 7)	−0.113	0.616	ns	0.893	
Hospitalization days (8 to 15)	−0.373	0.596	ns	0.689	
Hospitalization days (>15)	−1.396	0.697	0.045	0.248	
(Constant)	−1.160	0.589	0.049	0.313	

Note. *B* = logistic coefficient; *S.E*. = standard error of estimate; *p* = significance; *Exp(B)* = exponentiated coefficient; *R*^2^ = assessment of interpretive power; ns = nonsignificant.

## Data Availability

The data presented in this study are available on request from the corresponding author.

## References

[1] Ho C. (2013). Optimal duration of exclusive breastfeeding. Int. J. Evid. Based. Healthc..

[2] Sankar M.J., Sinha B., Chowdhury R., Bhandari N., Taneja S., Martines J., Bahl R. (2015). Optimal breastfeeding practices and infant and child mortality: A systematic review and meta-analysis. Acta Paediatr. Int. J. Paediatr..

[3] Ip S., Chung M., Raman G., Chew P., Magula N., DeVine D., Trikalinos T., Lau J. (2007). Breastfeeding and Maternal and Infant Health Outcomes in Developed Countries.

[4] Horta B., Victora C. (2013). Short-Term Effects of Breastfeeding: A Systematic Review on the Benefits of Breastfeeding on Diarrhoea and Pneumonia Mortality.

[5] Bowatte G., Tham R., Allen K., Tan D., Lau M., Dai X., Lodge C. (2015). Breastfeeding and childhood acute otitis media: A systematic review and meta-analysis. Acta Paediatr. Int. J. Paediatr..

[6] Lodge C., Tan D., Lau M., Dai X., Tham R., Lowe A., Bowatte G., Allen K., Dharmage S. (2015). Breastfeeding and asthma and allergies: A systematic review and meta-analysis. Acta Paediatr. Int. J. Paediatr..

[7] Horta B.L., Loret De Mola C., Victora C.G. (2015). Breastfeeding and intelligence: A systematic review and meta-analysis. Acta Paediatr. Int. J. Paediatr..

[8] Victora C.G., Barros A.J.D. (2000). Effect of breastfeeding on infant and child mortality due to infectious diseases in less developed countries: A pooled analysis. Lancet.

[9] Victora C.G., Horta B.L., de Mola C.L., Quevedo L., Pinheiro R.T., Gigante D.P., Gonçalves H., Barros F.C. (2015). Association between breastfeeding and intelligence, educational attainment, and income at 30 years of age: A prospective birth cohort study from Brazil. Lancet Glob. Health.

[10] Horta B.L., Loret De Mola C., Victora C.G. (2015). Long-term consequences of breastfeeding on cholesterol, obesity, systolic blood pressure and type 2 diabetes: A systematic review and meta-analysis. Acta Paediatr. Int. J. Paediatr..

[11] Amitay E.L., Keinan-Boker L. (2015). Breastfeeding and childhood leukemia incidence: A meta-analysis and systematic review. JAMA Pediatr..

[12] Dieterich C.M., Felice J.P., O’Sullivan E., Rasmussen K.M. (2013). Breastfeeding and Health Outcomes for the Mother-Infant Dyad. Pediatr. Clin. N. Am..

[13] Chowdhury R., Sinha B., Sankar M.J., Taneja S., Bhandari N., Rollins N., Bahl R., Martines J. (2015). Breastfeeding and maternal health outcomes: A systematic review and meta-analysis. Acta Paediatr. Int. J. Paediatr..

[14] González-Jiménez E., García P.A., Aguilar M.J., Padilla C.A., Álvarez J. (2014). Breastfeeding and the prevention of breast cancer: A retrospective review of clinical histories. J. Clin. Nurs..

[15] Marshall N.E., Abrams B., Barbour L.A., Catalano P., Christian P., Friedman J.E., Hay W.W., Hernandez T.L., Krebs N.F., Oken E. (2022). The importance of nutrition in pregnancy and lactation: Lifelong consequences. Am. J. Obstet. Gynecol..

[16] North K., Gao M., Allen G., Lee A.C. (2022). Breastfeeding in a Global Context: Epidemiology, Impact, and Future Directions. Clin. Ther..

[17] Hicks S.D., Confair A., Warren K., Chandran D. (2022). Levels of Breast Milk MicroRNAs and Other Non-Coding RNAs Are Impacted by Milk Maturity and Maternal Diet. Front. Immunol..

[18] Lokossou G.A.G., Kouakanou L., Schumacher A., Zenclussen A.C. (2022). Human Breast Milk: From Food to Active Immune Response With Disease Protection in Infants and Mothers. Front. Immunol..

[19] Gila-Díaz A., Carrillo G.H., de Pablo Á.L.L., Arribas S.M., Ramiro-Cortijo D. (2020). Association between maternal postpartum depression, stress, optimism, and breastfeeding pattern in the first six months. Int. J. Environ. Res. Public Health.

[20] Bartick M.C., Schwarz E.B., Green B.D., Jegier B.J., Reinhold A.G., Colaizy T.T., Bogen D.L., Schaefer A.J., Stuebe A.M. (2017). Suboptimal breastfeeding in the United States: Maternal and pediatric health outcomes and costs. Matern. Child Nutr..

[21] Bartick M.C., Jegier B.J., Green B.D., Schwarz E.B., Reinhold A.G., Stuebe A.M. (2017). Disparities in Breastfeeding: Impact on Maternal and Child Health Outcomes and Costs. J. Pediatr..

[22] Ogbo F.A., Eastwood J., Page A., Arora A., McKenzie A., Jalaludin B., Tennant E., Miller E., Kohlhoff J., Noble J. (2017). Prevalence and determinants of cessation of exclusive breastfeeding in the early postnatal period in Sydney, Australia. Int. Breastfeed. J..

[23] World Health Organisation (2020). The Best Start in Life Breastfeeding for the Prevention of Noncommunicable Diseases and the Achievement of the Sustainable Development Goals in the WHO European Region Moscow Russian Federation. https://iris.who.int/handle/10665/354398?fbclid=IwAR079sOMeOtYrh4HyXidXvbYdlwEiZ908X5JL5QbbQ3V4dXxTH8bw7aao0E.

[24] Sayres S., Visentin L. (2018). Breastfeeding: Uncovering barriers and offering solutions. Curr. Opin. Pediatr..

[25] Gianni M.L., Bettinelli M.E., Manfra P., Sorrentino G., Bezze E., Plevani L., Cavallaro G., Raffaeli G., Crippa B.L., Colombo L. (2019). Breastfeeding difficulties and risk for early breastfeeding cessation. Nutrients.

[26] Morrison A.H., Gentry R., Anderson J. (2019). Mothers’ Reasons for Early Breastfeeding Cessation. MCN Am. J. Matern. Nurs..

[27] Takahashi K., Ganchimeg T., Ota E., Vogel J.P., Souza J.P., Laopaiboon M., Castro C.P., Jayaratne K., Ortiz-Panozo E., Lumbiganon P. (2017). Prevalence of early initiation of breastfeeding and determinants of delayed initiation of breastfeeding: Secondary analysis of the WHO Global Survey. Sci. Rep..

[28] Revheim I., Balthasar M.R., Akerkar R.R., Stangenes K.M., Almenning G., Nygaard E., Markestad T., Øverland S., Roelants M., Juliusson P.B. (2023). Trends in the prevalence of breastfeeding up to 6 months of age using structured data from routine child healthcare visits. Acta Paediatr. Int. J. Paediatr..

[29] Penugonda A., Rajan R., Lionel A., Kompithra R., Jeyaseelan L., Mathew L. (2022). Impact of exclusive breast feeding until six months of age on common illnesses: A prospective observational study. J. Fam. Med. Prim. Care.

[30] Mena-Tudela D., Soriano-Vidal F.J., Vila-Candel R., Quesada J.A., Martínez-Porcar C., Martin-Moreno J.M. (2023). Is Early Initiation of Maternal Lactation a Significant Determinant for Continuing Exclusive Breastfeeding up to 6 Months?. Int. J. Environ. Res. Public Health.

[31] WHO, UNICEF (2003). Global Strategy for Infant and Young Child Feeding.

[32] Eidelman A.I. (2012). Breastfeeding and the use of human milk: An analysis of the American academy of pediatrics 2012 breastfeeding policy statement. Breastfeed. Med..

[33] Perrine C.G., Scanlon K.S., Li R., Odom E., Grummer-Strawn L.M. (2012). Baby-Friendly hospital practices and meeting exclusive breastfeeding intention. Pediatrics.

[34] Kozhimannil K.B., Jou J., Attanasio L.B., Joarnt L.K., McGovern P. (2014). Medically complex pregnancies and early breastfeeding behaviors: A retrospective analysis. PLoS ONE.

[35] McNestry C., Killeen S.L., Crowley R.K., McAuliffe F.M. (2023). Pregnancy complications and later life women’s health. Acta Obstet. Gynecol. Scand..

[36] Hediye A., Korkmaz N. (2005). The physical and psychological problems of hospitalized high-risk pregnant women in partial bed rest. Perinat. J..

[37] Rodrigues P.B., Zambaldi C.F., Cantilino A., Sougey E.B. (2016). Particularidades da gravidez de alto risco como fatores para o desenvolvimento de sofrimento mental. Trends Psychiatry Psychother..

[38] Kozhimannil K.B., Arcaya M.C., Subramanian S.V. (2014). Maternal Clinical Diagnoses and Hospital Variation in the Risk of Cesarean Delivery: Analyses of a National US Hospital Discharge Database. PLoS Med..

[39] Diaz Rozett H., Garcia Fragoso L. (2010). Prenatal breastfeeding intentions in a group of women with high risk pregnancies. Bol. Asoc. Medica P. R..

[40] Ahishakiye J., Bouwman L., Brouwer I.D., Vaandrager L., Koelen M. (2020). Prenatal infant feeding intentions and actual feeding practices during the first six months postpartum in rural Rwanda: A qualitative, longitudinal cohort study. Int. Breastfeed. J..

[41] Manstead A.S., Proffitt C., Smart J.L. (1983). Predicting and understanding mothers’ infant-feeding intentions and behavior: Testing the theory of reasoned action. J. Pers. Soc. Psychol..

[42] Parker M.G., Hwang S.S., Forbes E.S., Colvin B.N., Brown K.R., Colson E.R. (2020). Use of the theory of planned behavior framework to understand breastfeeding decision-making among mothers of preterm infants. Breastfeed. Med..

[43] Dagla M., Mrvoljak-Theodoropoulou I., Karagianni D., Dagla C., Sotiropoulou D., Kontiza E., Kavakou A.T., Antoniou E. (2021). Women’s mental health as a factor associated with exclusive breastfeeding and breastfeeding duration: Data from a longitudinal study in Greece. Children.

[44] Dagla M., Mrvoljak-Theodoropoulou I., Vogiatzoglou M., Giamalidou A., Tsolaridou E., Mavrou M., Dagla C., Antoniou E. (2021). Association between breastfeeding duration and long-term midwifery-led support and psychosocial support: Outcomes from a greek non-randomized controlled perinatal health intervention. Int. J. Environ. Res. Public Health.

[45] Gartner L.M., Morton J., Lawrence R.A., Naylor A.J., O’Hare D., Schanler R.J., Eidelman A.I., American Academy of Pediatrics Section on Breastfeeding (2005). Breastfeeding and the use of human milk. Pediatrics.

[46] Scime N.V., Metcalfe A., Nettel-Aguirre A., Tough S.C., Chaput K.H. (2021). Association of prenatal medical risk with breastfeeding outcomes up to 12 months in the All Our Families community-based birth cohort. Int. Breastfeed. J..

[47] Lyons K.E., Ryan C.A., Dempsey E.M., Ross R.P., Stanton C. (2020). Breast milk, a source of beneficial microbes and associated benefits for infant health. Nutrients.

[48] Stuebe A.M., Schwarz E.B. (2010). The risks and benefits of infant feeding practices for women and their children. J. Perinatol..

[49] Donath S., Amir L. (2003). Relationship between prenatal infant feeding intention and initiation and duration of breastfeeding: A cohort study. Acta Paediatr..

[50] Declercq E., Labbok M.H., Sakala C., O’Hara M.A. (2009). Hospital practices and women’s likelihood of fulfilling their intention to exclusively breastfeed. Am. J. Public Health.

[51] Cordero L., Valentine C.J., Samuels P., Giannone P.J., Nankervis C.A. (2012). Breastfeeding in women with severe preeclampsia. Breastfeed. Med..

[52] Cordero L., Gabbe S.G., Landon M.B., Nankervis C.A. (2013). Breastfeeding initiation in women with gestational diabetes mellitus. J. Neonatal. Perinatal. Med..

[53] Cordero L., Thung S., Landon M.B., Nankervis C.A. (2014). Breast-feeding initiation in women with pregestational diabetes mellitus. Clin. Pediatr..

[54] Cordero L., Oza-Frank R., Landon M.B., Nankervis C.A. (2015). Breastfeeding Initiation among Macrosomic Infants Born to Obese Nondiabetic Mothers. Breastfeed. Med..

[55] Call C.C., Hawkins M.S., Shah V.K., Frank D., Niemi S., Jouppi R.J., Ferguson E., Conlon R.P.K., Levine M.D. (2024). A longitudinal investigation of breastfeeding planning, initiation, and duration among individuals with pre-pregnancy overweight or obesity. Appetite.

[56] DiGirolamo A., Thompson N., Martorell R., Fein S., Grummer-Strawn L. (2005). Intention or experience? Predictors of continued breastfeeding. Health Educ. Behav..

[57] Thulier D. (2010). A call for clarity in infant breast and bottle-feeding definitions for research. JOGNN-J. Obstet. Gynecol. Neonatal Nurs..

[58] Hundalani S.G., Irigoyen M., Braitman L.E., Matam R., Mandakovic-Falconi S. (2013). Breastfeeding among inner-city women: From intention before delivery to breastfeeding at hospital discharge. Breastfeed. Med..

[59] Gross S.M., Resnik A.K., Nanda J.P., Cross-Barnet C., Augustyn M., Kelly L., Paige D.M. (2011). Early postpartum: A critical period in setting the path for breastfeeding success. Breastfeed. Med..

[60] Cordero L., Stenger M.R., Landon M.B., Nankervis C.A. (2021). Breastfeeding initiation among women with preeclampsia with and without severe features. J. Neonatal. Perinatal. Med..

[61] Strapasson M.R., Ferreira C.F., Ramos J.G.L. (2018). Feeding practices in the first 6 months after delivery: Effects of gestational hypertension. Pregnancy Hypertens..

[62] Huang R., Wan Y., Yao X., Wang H., Cai C., Xu Y., Jiang H. (2023). Predictive factors of exclusive breastfeeding attrition at Week 6 post-partum among mothers of preterm infants based on the theory of planned behaviour. Matern. Child Nutr..

[63] Stuebe A.M., Bonuck K. (2011). What predicts intent to breastfeed exclusively? breastfeeding knowledge, attitudes, and beliefs in a diverse urban population. Breastfeed. Med..

[64] Tenfelde S., Finnegan L., Hill P.D. (2011). Predictors of Breastfeeding Exclusivity in a WIC Sample. JOGNN-J. Obstet. Gynecol. Neonatal Nurs..

[65] Nommsen-rivers L.A., Mastergeorge A.M., Hansen R.L., Cullum A.S., Dewey K.G. (2009). Doula care, early breastfeeding outcomes, and breastfeeding status at 6 weeks postpartum among low-income primiparae. JOGNN-J. Obstet. Gynecol. Neonatal Nurs..

